# Community-based environmental management for malaria control: evidence from a small-scale intervention in Dar es Salaam, Tanzania

**DOI:** 10.1186/1475-2875-8-57

**Published:** 2009-04-08

**Authors:** Marcia C Castro, Atsuko Tsuruta, Shogo Kanamori, Khadija Kannady, Sixbert Mkude

**Affiliations:** 1 Department of Global Health and Population, Harvard School of Public Health, 665 Huntington Avenue, Bldg. I, Room 1113, Boston, MA, 02115, USA; 2 Senior Researcher, International Development Center of Japan, Tokyo, Japan; 3 Department of International Community Health, Graduate School of Medicine, The University of Tokyo, Tokyo, Japan; 4 City Medical Office of Health, Dar es Salaam City Council, Dar es Salaam, Tanzania; 5 National Malaria Control Programme, Dar es Salaam, Tanzania

## Abstract

**Background:**

Historically, environmental management has brought important achievements in malaria control and overall improvements of health conditions. Currently, however, implementation is often considered not to be cost-effective. A community-based environmental management for malaria control was conducted in Dar es Salaam between 2005 and 2007. After community sensitization, two drains were cleaned followed by maintenance. This paper assessed the impact of the intervention on community awareness, prevalence of malaria infection, and *Anopheles *larval presence in drains.

**Methods:**

A survey was conducted in neighbourhoods adjacent to cleaned drains; for comparison, neighbourhoods adjacent to two drains treated with larvicides and two drains under no intervention were also surveyed. Data routinely collected by the Urban Malaria Control Programme were also used. Diverse impacts were evaluated through comparison of means, odds ratios (OR), logistic regression, and time trends calculated by moving averages.

**Results:**

Individual awareness of health risks and intervention goals were significantly higher among sensitized neighbourhoods. A reduction in the odds of malaria infection during the post-cleaning period in intervention neighbourhoods was observed when compared to the pre-cleaning period (OR = 0.12, 95% CI 0.05–0.3, p < 0.001). During the post-cleaning period, a higher risk of infection (OR = 1.7, 95% CI 1.1–2.4, p = 0.0069) was observed in neighbourhoods under no intervention compared to intervention ones. Eighteen months after the initial cleaning, one of the drains was still clean due to continued maintenance efforts (it contained no waste materials and the water was flowing at normal velocity). A three-month moving average of the percentage of water habitats in that drain containing pupae and/or *Anopheles *larvae indicated a decline in larval density. In the other drain, lack of proper resources and local commitment limited success.

**Conclusion:**

Although environmental management was historically coordinated by authoritarian/colonial regimes or by industries/corporations, its successful implementation as part of an integrated vector management framework for malaria control under democratic governments can be possible if four conditions are observed: political will and commitment, community sensitization and participation, provision of financial resources for initial cleaning and structural repairs, and inter-sectoral collaboration. Such effort not only is expected to reduce malaria transmission, but has the potential to empower communities, improve health and environmental conditions, and ultimately contribute to poverty alleviation and sustainable development.

## Background

The World Health Organization (WHO) defines environmental management (EM) for vector control as "*The planning, organization, carrying out and monitoring of activities for the modification and/or manipulation of environmental factors or their interaction with man with a view to preventing or minimizing vector propagation and reducing man-vector-pathogen contact*" [1]. EM is not a replacement of other interventions, but one of several optional components that will make up an integrated vector management (IVM) approach in a vector control programme [2-4]. Indeed, mathematical models of malaria transmission indicated that integrated control programs that combine multiple interventions are more likely to succeed than programs that promote only one intervention [5,6]. Historically, EM has brought important achievements in malaria control and overall improvements of health conditions [7-11]. It made possible the construction of the Panama canal, the rubber production in Malaysia, and copper mining in Zambia, to name a few. Unsuccessful EM interventions were largely a consequence of inadequate planning and implementation (e.g., intervention in Mian Mir, India) [12]. The golden age of EM was prior to World War II and this coincided with authoritarian/colonial regimes or was related to industries/corporations. In such settings, human, technical, and financial resources were not a constraint. This is a sharp contrast with current realities in malaria-endemic countries, where resources are scarce and inter-sectoral (e.g., public health, engineering, and urban planning) cooperation and coordination is difficult. The post-war development of powerful chemical tools has contributed dramatically to this fragmentation [13,14]. Therefore, the challenge is to show how to currently promote sustainable and cost-effective EM within an IVM approach run by democratic governments.

Some past EM efforts were discontinued with the onset of the Eradication Campaign in the late 1950s [13]. The focus on indoor residual spraying (IRS) with DDT was supported by mathematical models of malaria that indicated the potential impact of reducing the longevity of mosquitoes, *p*, on the basic reproduction rate, *R*_*0 *_– a measure of transmission potential that indicates the expected number of secondary infections caused by the introduction of an infectious individual in a susceptible population [15]. EM, however, focuses on breeding source reduction and therefore impacts mosquito density, *m *(or the number of mosquitoes per person) [15,16]. Mathematically, a reduction in *m *also impacts *R*_*0*_, but not with the same magnitude as a reduction in *p*, assuming other parameters are held constant. The impact of past control efforts in Africa has been empirically assessed and suggested that EM did reduce mosquito biting rate, but also increased mosquito mortality because of longer time spent foraging for oviposition after source reduction [17]. Therefore, EM has the potential to impact both *p *and *m*, resulting in reductions in *R*_*0*_, and declines in the entomologic inoculation rate, EIR – a measure of transmission intensity that indicates the number of infective bites per person per year [6,17].

In Dar es Salaam, Tanzania, EM activities commenced during the colonial period and were continued briefly after independence [18-22]. Positive achievements suffered a major setback in 1972, when adverse economic conditions resulted in deterioration of the health system. Maintenance of drains was nonexistent; water flow was blocked by silt, vegetation, and waste, favouring the occurrence of flooding, and offering ideal conditions for mosquito breeding [23]. Meanwhile, rapid urban growth characterized by lack of access to sanitation and safe drinking-water, precarious housing, overcrowding, and inefficient waste collection added yet another layer of challenges for vector control. It is estimated that in Dar es Salaam 70% of the population lives in unplanned settlements [24]. Most are not accessible by motor vehicles, hampering waste collection. Currently, approximately 50% of all refuse daily generated is not collected; it accumulates on the ground, eventually flowing into drains and rivers. The network of drains in Dar es Salaam represents a major risk for malaria transmission: in 2005–06, 47% of all water habitats with pupae and/or *Anopheles *larvae mapped in 15 wards of the city were drains.

**Figure 1:**
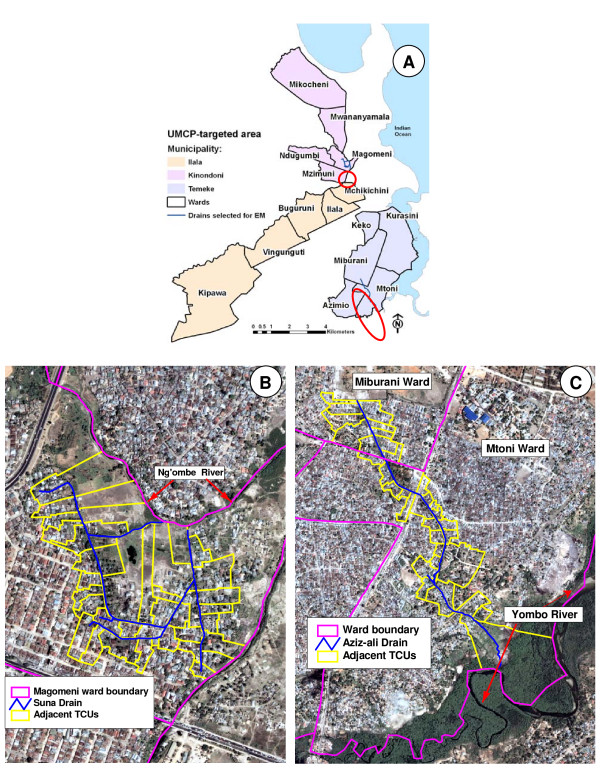
**Urban Malaria Control Programme (UMCP) targeted area and drains selected for pilot community-based environmental management intervention**. A. UMCP-targeted wards where a survey to assess drain conditions was conducted. Two drains selected for a pilot community-based environmental management intervention are circled in red. B. Suna Drain, located in Magomeni ward. C. Aziz-ali Drain, located in Mtoni and Miburani wards. In both B and C, the background is a Quickbird satellite image taken in 2002. Since that year, significant house construction occurred near Magomeni ward.

A partnership between the Japan International Cooperation Agency (JICA), the National Malaria Control Programme (NMCP), and the Urban Malaria Control Programme (UMCP) planned and implemented a pilot intervention of community-based EM of *Anopheles *breeding sites in Dar es Salaam in 2005–07 [25]. The NMCP expected to obtain scientific evidence of the feasibility and impact of EM, and specific operational protocols of EM implementation and community sensitization that could guide future malaria vector control as part of an IVM approach [2,3]. The intervention comprised three phases: (i) initial assessment of drains, (ii) community sensitization and drain cleaning, and (iii) evaluation. Phase I (planned and executed between June 2005 and December 2006) aimed at providing a comprehensive description of drains located in wards targeted by the UMCP (Figure 1). A total of 107.6 km of drain segments were surveyed: 51% contained solid waste and sand, 13% had normal water flow, 1% had on-going maintenance efforts, 38% had history of flooding, and in 24% the water was stagnant [26]. After this initial assessment, two drains (Figure 1) were selected for a pilot EM intervention (planned and executed between November 2006 and March 2007). Selection criteria were: (i) located in areas without larviciding spraying; (ii) high potential for *Anopheles *breeding; (iii) connected to a river; (iv) history of flooding; (v) small ward; (vi) age of the drain; and (vii) not part of an intricate network of connecting drains. The first, Suna, located in Magomeni ward, Kinondoni Municipality, extends to 2.1 km flowing into Ng'ombe River, and runs through administrative units (called tencell units – TCUs) with an estimated population of 35 thousand people. The second, Aziz-ali, located in Mtoni and Miburani wards, Temeke Municipality, extends 1.7 km flowing into Yombo River, and runs through TCUs with an estimated population of 60 thousand [27].

Drain cleaning activities were planned in a participatory manner involving community members, and city, municipal and ward representatives; no work was executed before community evaluation. Initial cleaning was performed by a contractor, and 90% of the hired workforce lived in the community. Activities also included grass cutting and minor repairs (e.g., slab replacement). Aziz-ali drain was cleaned in January 2007, and Suna drain in February/March 2007. The latter required the use of an excavator due to the poor conditions of the river. Community sensitization aimed at providing information on malaria transmission and EM activities. It was conducted through community leaders' seminars (18 seminars, 550 people), mass meetings (10 meetings, 4,000 people), and house-to-house visits (85,042 people from March-August 2007). A comprehensive description of the community-based EM activity protocol was assembled in a manual, which will serve as a guide for future EM work in Tanzania [27]. Maintenance of drains cleaned in Phase II was transferred to the municipalities in November 2007.

The underlying hypothesis is that by conducting EM in a community participatory manner, followed by continued maintenance, there would be an increase in local awareness, a reduction in flooding during the rainy season, the flow of water in the drain would be restored, and the number of *Anopheles *breeding sites reduced. In this paper, an evaluation of the pilot community-based EM intervention in Dar es Salaam is presented, including an assessment of the impact that EM activities had on the prevalence of malaria infection in the immediate neighbourhood where drains were cleaned, and an appraisal of the presence of *Anopheles *larvae in the drains. Also, the role of sensitization on community awareness and willingness to take part on EM activities was assessed. Finally, administrative procedures and budget allocation for EM maintenance activities in each municipality, which can plausibly contribute to the success and sustainability of the intervention, were investigated.

## Methods

### Data sources

To assess the impact of the EM intervention, a cross-sectional study was conducted in six sites between July 2007 and July 2008. Each site included TCUs neighbouring a drain. Selection of sites aimed at facilitating the comparison of three different scenarios: EM (two drains cleaned in Phase II), LVC – use of larviciding since March 2006 (two sites), and NO – no intervention (two sites). Selection criteria for the last two scenarios were: (i) drain with high potential for *Anopheles *breeding; (ii) history of flooding; (iii) not part of an intricate network of connecting drains; and (iv) age of the drain. Although sites that are perfect replicates regarding social and environmental characteristics cannot be selected, all study sites had unpaved roads, equivalent house quality, faced equal waste collection challenges, and time lived in the house averaged eight years. Baseline data collected by the UMCP [28] prior to the EM intervention indicated that the prevalence of malaria infection in TCUs surrounding selected drains in all six sites were similar and ranged from 10.2–12% among all age groups. In order to show a 60% reduction in infection after the EM, the intervention required a sample size of 259–309 subjects, considering a 95% significance level and 80% power. A 300-sample size in each site was set up as a target.

Sites were visited every other month to account for rainfall seasonality. For each survey round, 75 houses were spatially randomly selected based on a 2002 Quickbird satellite image. In each house, four people were invited to participate in the survey (two adults and two children), according to the following criteria: (i) adults: older than 15 and younger than 55, and lived in the house for more than one year; if more than two candidates, the following decisive factors were used: head of the household, lived in the house for the longer period of time, alphabetical order (first name); (ii) children: aged 15 or younger, and lived in the house for more than one year; if more than two candidates, the following decisive factors were used: youngest child, lived in the house for the longer period of time, alphabetical order (first name). A total of 9,070 interviews were performed (Table 1): 51% adults and 49% children (18% below 5 years and 31% aged 5–15). Operational problems during survey round 1 required further data collection of half of the sample in July 2008.

**Table 1:** Participants in the evaluation survey by site type and round of interview.

Survey Round	EM		LVC		NO		Total
		
	Adults	Children	Adults	Children	Adults	Children	
1 – Jul/Oct 2007	299	299	143	130	290	1	1,162
2 – Nov/Dec 2007	300	207	204	201	301	300	1,513
3 – Jan/Feb 2008	300	301	393	394	300	303	1,991
4 – Mar/Apr 2008	298	303	297	300	294	301	1,793
5 – May/Jun 2008	299	295	215	301	297	304	1,711
6 – Jul 2008	143	159	150	150	139	159	900

Total	1,639	1,564	1,402	1,476	1,621	1,368	9,070

Two interviewers left the study without notice in survey round 1, which delayed the planned schedule. In some houses there were not 4 individuals matching selection criteria.

Individual human subjects were informed about survey goals and invited to participate. Upon agreement, informed consents were signed, a questionnaire answered, and finger prick blood samples collected. Three different questionnaires were developed for each site type, but a core set of questions related to demographic characteristics, recent malaria infection, perception regarding presence of waste in drains, and willingness to participate in EM community efforts were common to all. Malaria parasites were identified by species using thin smears [29]. The Medical Research Coordination Committee of the National Institute of Medical Research in Tanzania and the Tanzanian Commission of Science and Technology provided ethical clearance for the study.

To assess prevalence of malaria infection and larval presence prior to July 2007, larval and parasitological data routinely collected by the UMCP [28] were used. Information on costs of EM activities was obtained from JICA and from the municipalities. The latter also provided data on administrative procedures for EM maintenance activities. Information on the phased use of larviciding was obtained, which might affect the temporal trend in prevalence of infection: in April 2008 the UMCP started to spray larvicides in all six survey sites. Lastly, data on monthly rainfall were obtained from the Tanzania Meteorological Agency.

### Statistical analysis

Survey data allowed the assessment of awareness and willingness to participate in EM activities. Differences in proportions of people aware of control interventions in EM and LVC sites were tested. For all three site types, differential attitudes towards participation in EM initiatives were appraised.

Survey and UMCP data were combined to generate a database comprising information on prevalence of infection for 2005–2008 for all six study sites. Information on rainfall was added to control for the seasonal pattern of malaria transmission. A time series of prevalence of malaria infection rates for the study sites was assembled, and Clopper-Pearson binomial confidence intervals for the rates were calculated. The variable age was grouped into three categories (below 5, 5–15, and above 15) to reflect differences in malaria risk by age. In order to assess the impact of the EM intervention in the prevalence of infection, unadjusted odds ratio (OR) of malaria infection before and after drain cleaning, and in each site type after cleaning were calculated. OR adjusted for rainfall, bed net use, age, and larviciding use after April 2008 were also computed. A stepwise logistic regression (backward selection, p = 0.2) for the presence of malaria infection including terms for period of intervention, bed net use, age, rainfall, larviciding use, type of site, and an interaction term for age-time of intervention was calculated to test for heterogeneity in the effect of EM activities.

Routine UMCP larval data (January 2005–December 2007) were used to obtain a monthly time series of the percentage of water habitats found in drains targeted with EM that contained pupae and/or *Anopheles *larvae. A three-month moving average was used to extract the time trend observed in each drain, and the slope (and confidence interval) of the trend after cleaning was calculated.

## Results

The analyses show significant impacts of community sensitization. In EM sites, 96% (95% CI 95–97) of the residents knew about cleaning activities, mostly communicated in meetings or by TCU leaders, 56% (95% CI 54–59). In LVC areas, where larviciding commenced in March 2006, 88% (95% CI 86–90) were aware of the control efforts, but mainly because the sprayman was seen at work (64%; 95% CI 61–66). The declared willingness to participate in efforts to clean drains did not reveal significant differences for LVC and NO sites (average of 75%). In EM sites, however, 23% reported using tools available at the ward office to clean the drain, while 52% indicated no use due to lack of proper skills, and 15% were not willing to participate unless financial incentives were provided. Although more than 200 community members worked voluntarily in group efforts organized during initial cleaning, unpaid individual effort on a continued basis is not feasible, as previously reported [30]. Results also indicate that sensitization improved community's perception on the benefits of drain cleaning. Significant differences between LVC and EM sites raise questions about people's ability to respond to health risks at individual and community levels: while 61% (95% CI 59–64) of those targeted by sensitization (EM sites) believed that drain cleaning could reduce the number of mosquitoes, in LVC only 30% (95% CI 28–33) had this perception.

Regarding the prevalence of malaria infection, a significant reduction in the unadjusted OR of infection during the post-cleaning period (after January 2007) was observed in EM sites, when compared to the pre-intervention period (2005–2006), 0.23 (95% CI 0.14–0.38, p < 0.001). When adjusted for age, rainfall, bed net use, and larviciding spray after April 2008 the OR were 0.12 (95% CI 0.05–0.3, p < 0.001). Such impact compares in magnitude with positive results of EM summarized by a recent systematic review and meta-analyses [11]. A decline in the risk of infection was also observed in LVC sites, but not significantly different from that recorded in EM sites. The additional effect of larviciding use in EM sites during April-July 2008 was not assessed, since these were dry months and therefore did not capture seasonal effects in malaria risk. Comparison of sites during the post-intervention period also suggested a positive impact of EM activities: while unadjusted OR of malaria infection did not show significant differences between EM, LVC and NO sites, when adjusted for age, rainfall, bed net use, and larviciding spray after March 2008, NO sites had a higher risk of infection (OR = 1.7, 95% CI 1.1–2.4, p = 0.0069) when compared to EM sites.

Stepwise multivariate logistic regression revealed higher OR among children aged 5–15 (OR = 2.3, 95% CI 1.6–3.3, p < 0.001) than those younger than five years (OR= 1.6, 95% CI 1.0–2.4, p = 0.047) compared to adults. Although this age pattern had been reported in other areas [31], it may be a result of the better representation of older children in the sample: 64% (n = 2,819) of all children surveyed were aged 5–15. No significant differences regarding bed net use among these two age groups were observed, and the higher OR among older children was not significant in univariate analysis. The addition of an interaction term in the logistic model showed no evidence of differences in the effects of EM activities by age.

**Figure 2:**
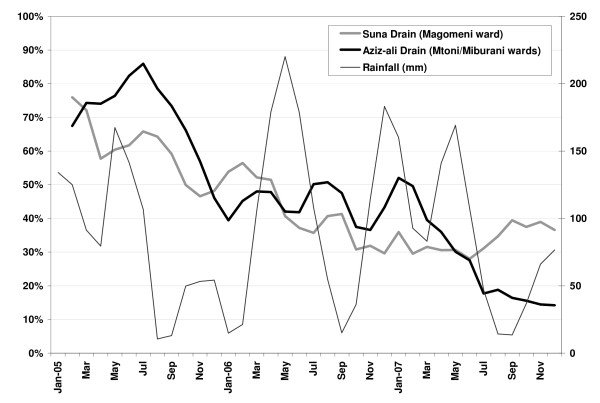
**Rainfall and temporal trend of the percentage of water habitats in drains targeted with community-based environmental management that contained pupae and/or *Anopheles *larvae from 2005 to 2007**. Data are from the Tanzania Meteorological Agency (Rainfall) and the UMCP routine larval inspection. The time trend was obtained by calculating a 3-month moving average of the percentage of water habitats in drains that contained pupae and/or *Anopheles *larvae from Jan/2005 to Dec/2007.

Regarding the presence of larvae, a three-month moving average of the percentage of water habitats in drains containing pupae and/or *Anopheles *larvae showed that larval density in Aziz-ali drain declined after EM activities (January 2007), while Suna drain did not improve (Figure 2). Slope of the temporal trends after cleaning confirmed the contrast: 0.7 (95% CI 0.1–1.3) for Aziz-ali, and -3.6 (95% CI -[4.5-2.8]) for Suna. On-site inspections reported excellent conditions in Aziz-ali, while Suna resembled conditions observed during the pre-cleaning period. This raises the question: was the lack of impact in larval density in Suna drain accompanied by unchanging or increasing prevalence of infection? Results show that the prevalence of infection after cleaning was not significantly different in these two drains, but bed net use significantly increased in the Suna area from 86% (95% CI 82–90) before the EM intervention to 98.7% (95% CI 98–99) afterwards, the largest bed net use among all studied sites throughout the survey period.

With respect to costs, US$ 30,800 was spent during the initial massive cleaning in Temeke (US$ 18/meter), and US$ 23,000 (US$ 11/meter) in Kinondoni. These costs could have been higher if major structural repairs were necessary; maintenance, however, can be performed at very low costs [32,33]. Each municipality has the autonomy to allocate funds, and an important difference was observed in the maintenance protocol. Temeke municipality assigned US$ 5,150 (US$ 3/meter) per year for maintenance activities (personnel and equipment) under the Council Comprehensive Health Plan (CCHP); 8 people were hired in the community (community owned resource person – CORP) for the task, working 18 days per month, with a small payment of US$ 3/day. Kinondoni municipality, however, allocated only US$ 2,536 (US$ 1.5/meter), and placed the activity under the responsibility of the Engineering Department; funds were exhausted in six months, and lack of links between the health and the engineering sectors, coupled with poor commitment, resulted in unsuccessful activities in Kinondoni. Combining initial cleaning and maintenance costs in Temeke over one year, the cost per resident in TCUs adjacent to the drain was US$ 0.60. However, this indicator did not account for costs related to the community sensitization activities, capital costs (e.g., vehicles and building space), some recurrent costs (such as operating costs of vehicles and buildings), and short-term experts/advisors. A comprehensive cost analysis and cost-effective analysis of the EM pilot intervention is currently being undertaken, utilizing standard methodology [34-36]. Results will provide much needed evidence regarding the cost-effectiveness of community-based EM interventions undertaken in urban Africa, and will facilitate comparison between EM and other vector control strategies.

In a scenario where governmental sectors are often competing for scarce financial resources, maintenance EM activities launched by the health sector are seen solely as a health intervention. Implications of these activities to other sectors are neglected and cooperative initiatives inexistent. The unsuccessful maintenance efforts in Kinondoni may reflect this problem – the importance of EM activities was overlooked, resulting in under allocation of resources and precarious monitoring. Alternative institution arrangements that recognize the importance of multidisciplinary work have the potential to mitigate this problem [37], as discussed next.

## Discussion

Historically, EM has brought important achievements in malaria control and overall improvement of health conditions [7-10]. A recent community-based EM initiative in Dar es Salaam aimed at establishing protocols for EM implementation and at providing scientific evidence of its feasibility. Results showed an enhancement in people's perception about the risk of dirty and blocked drains, 88% decrease in the odds of malaria infection, and a decline in the larval/pupae presence in one of the treated drains. These results were observed during an evaluation assessment commenced 4–6 months after the initial drain cleaning, and conducted over a 13-month period.

The choice of outcome indicators needs further discussion. While there is evidence that EM can impact both the density and the longevity of mosquitoes, and therefore reduce the EIR and *R*_*0 *_[6,17], translation of these impacts into disease outcomes, such as the prevalence of malaria infection, is not straightforward [38]. Although the literature on mathematical models of malaria is extensive and still growing, a comprehensive (and therefore multidisciplinary) framework that is spatially explicit, able to represent transmission at the local level, and that relates different outcome indicators is yet to be designed. In Dar es Salaam, the EIR observed over a period of two years (April 2005 to March 2007) was 1.33 infectious bites per person per year for the average resident – 1.00, 0.13 and 0.20 infectious bites per person per year for *An. gambiae*, *An. funestus *and *An. coustani*, respectively [39]. The prevalence of malaria infection was weakly related to locally measured EIR, except for the age group 0–5 years of age [39]. Other non-entomological factors impact levels of prevalence, and further research is needed in order to improve understanding of how these factors mediate the relationship between EIR and prevalence. The pilot community-based EM intervention did not collect information on EIR because of operational difficulties. Routine UMCP monitoring activities indicated that available mosquito traps were not sufficiently sensitive to low *Anopheles *density observed in Dar es Salaam, and therefore human landing catch (HLC) was the most suitable approach for adult mosquito surveys [40]. Among the 330 houses routinely monitored by the UMCP through HLC, only one was located within the TCUs adjacent to Suna drain and only three within TCUs adjacent to Aziz-ali drain. Therefore, there was not sufficient information to represent the intensity of malaria transmission in the EM sites prior to the intervention. In addition, considering the very low levels of EIR observed in the wards monitored by the UMCP, a large sample size would be required in order to detect significant variations in the intensity of malaria transmission. Also, malaria transmission in urban areas is likely to be focal since the dispersal of *Anopheles *in areas with high human population density tends to be no more than a few hundred meters from breeding sites [41]. Consequently, reducing the density of mosquitoes is expected to directly impact disease outcomes. Therefore, we chose to utilize the prevalence of malaria infection, controlling for the potential impact of rainfall, bed net use, age, and use of larviciding. Although these variables do not account for all possible factors that impact overall levels of prevalence, results from the analysis of all six selected sites (which had similar prevalence levels before EM activities) are expected to provide initial evidence of the impact of the pilot EM community-based intervention.

Considering the chosen outcomes, positive impacts following EM activities raise questions regarding the quality of data. Quality check was conducted on a 10% sample of blood slides at the Muhimbili University of Health and Allied Sciences – MUHAS (a centre of excellence in laboratory analysis), indicating a 94.5% specificity rate and 95.7% sensitivity rate. Cleaning of survey data was conducted at the end of every survey round. Less than 1% of the questionnaires had problems with missing or inconsistent answers, and there were no missing data on blood test results and age. Bias on the sample is unlikely, since selection of individuals in each house was made by the interviewers, and not determined by residents. With respect to larval data, quality has improved since 2006 [28]. Misclassification of water habitats is unlikely, since drains are distinctive features in the landscape.

Considering that data quality is not an issue, the analysis of the impact of EM activities raises two questions. First, can the successful EM activities in Aziz-ali drain be sustained over time? Commitment of medical officers in Temeke municipality facilitated proper and continued maintenance. A proposal to increase the budget for drain cleaning by CORPs in the next fiscal year has been submitted by the municipality (yet to be approved). Implementation of EM activities on a community-basis empower local residents, develops a sense of ownership, improve environmental responsibility among the population, and do not impose further constraints to the currently insufficient health staff [42]. However, the implementation of sensitization/education programmes needs to be conducted on a regular basis and with a larger coverage. For example, part of the waste currently removed from the Aziz-ali drain comes from the river; a result of improper disposal in other areas of the city. Therefore, programmes that inform the community and contribute to behaviour change to promote better and healthier environments are crucial [43,44]. Such programmes could be articulated in a collaborative manner between the government and varied institutions. For example, a partnership with the Dar es Salaam University (involving areas such as Engineering, Biology, Sociology, Anthropology, Economics, and Geography) could provide human resources for research, implementation, and monitoring of programmes, and stimulate capacity building of much needed professionals that could, in the future, be absorbed into health-related activities. Also, non-governmental organizations (NGOs), already very active in Tanzania [45], could work with the Dar es Salaam City Council and community leaders toward establishing a programme agenda with clear goals to be achieved and indicators to be monitored.

Regarding legal aspects, the current legislation qualifies as a public offense for the occupant of a plot to allow mosquito breeding sites to develop in their land [46]. Violations result in payment of fees starting at approximately US$ 625, depending on the gravity of the problem. Enforcement of the law, however, is far from ideal, but could be improved through sensitization/education programmes that increase local awareness and result in individual and collective (peer-pressure) behaviour change.

In addition, a critical issue for sustainability is the need to build bridges between sectors that currently tend to work in isolation, such as engineering, waste management, and public health [37]. Deficient waste management imperils the functioning of drains, contaminates groundwater [47], and limits positive achievements of a sensitization programme. A concerted effort fostering inter-sectoral linkages has the potential to address the challenges of a fast growing city and enhance the healthy conditions of its inhabitants. Those challenges involve sectors such as agriculture (irrigation practices that impact the number of aquatic habitats for mosquito breeding), transportation (road construction that favour the development of small and temporary breeding sites), energy (mining and hydroelectric projects that often increase mosquito density), urban planning (building construction and selection of new areas for settlement), waste management, and engineering. Mechanisms to foster inter-sectoral linkages are still at primitive stages. Yet, alternatives need to be evaluated. Engineers can work with health officials to devise approaches to reduce water habitats and improve housing conditions [48]. Several NGOs promote programmes that focus on installing fresh water and sanitation facilities, and training local personnel in the technology skills and maintenance. Potential positive health impacts of these programmes, however, are not registered in international health statistics, as there is no systematic connection between the NGOs and the health community [37]. The Tanzania Essential Health Interventions Project (TEHIP) was a major step toward repairing and maintaining health systems at the district level [49]. The addition of a multidisciplinary team of professionals (sanitary engineer, hydrologist, and urban planner) to the health system staff would maximize TEHIP's positive benefits by improving the quality and diversity of the available information for evidence-based planning [37,50]. Also, much of the construction and large-scale development projects do not properly assess and implement action to mitigate the negative impacts these projects can have on health [37,51,52]. There is a need to improve policies and legislation that require health impact assessments (HIAs) prior to the construction of such projects, and that enforce actions to guarantee that the health conditions of the impacted populations will not be deteriorated. Although development banks (e.g., World Bank) and some specific countries have imposed regulations that require environmental impact assessments, HIAs are often not addressed. Critical to the major goal of fostering inter-sectoral linkages, however, is the need to build local capacity. Currently, most malaria endemic countries lack the local expertise to establish a multidisciplinary team to plan, implement, monitor and evaluate control interventions. Professionals qualified as malaria engineers, which used to be part of the health sector staff until the early 1970s, are not available and new ones are not being trained. If HIAs were to become a requirement, the health sector would lack qualified personnel to evaluate them. Indeed, the need for capacity building and for inter-sectoral collaboration are two of the characteristics of an IVM framework [2,3].

The second question is: Can EM activities be scaled-up? Unplanned areas would certainly benefit from community-based EM activities. The operational procedures used in the pilot intervention have been documented and can be replicated to other areas [27]. However, a word of caution is needed. First, EM should not be implemented in isolation, but embedded in an IVM framework of vector control. Second, some drains require engineering work: inappropriate slope and structural erosion will hamper normal water flow even after massive cleaning (monitoring the demand for such work could be improved by the incorporation of an engineer to the NMCP team). In areas close to roads, drains have covered segments that will entail special cleaning machinery. Waste collection improvement is imperative to reduce refuse accumulation. That demands the acquisition of additional garbage trucks and handcarts. For example, Temeke municipality has five trucks, each able to perform four daily trips to the city dumping site, when in perfect conditions. Still, 63% of the total refuse generated daily remains uncollected. In such a scenario, maintenance activities could be undertaken by the city if proper coordination and commitment is in place, as observed in Aziz-ali drain. Non-expensive alternatives, such as the placement of sandbags in the banks of the drain to increase stability, can be undertaken with community involvement, as already observed in some locations. However, prior to maintenance, scaling-up EM activities will require resources for initial massive cleaning and structural repairs that the city is not likely to afford without donor support.

The National Malaria Medium Term Strategic Plan for 2008–2013 was developed by the Tanzania Ministry of Health and Social Welfare in line with the new call for malaria elimination. One of the strategic components of the plan is an IVM framework combining the use of insecticide-treated nets (ITNs), indoor residual spraying (IRS), EM, and larviciding. The country, however, lacks about 2/3 of the necessary financial resources to implement the strategic plan in its totality. The Millennium Development Goals (MDG) working group on malaria included EM as one of interventions for malaria vector control, and suggested the promotion of community participation as one of the critical issues for priority action [53]. Despite MDG's recommendations, funding for EM activities is scarce. The Global Fund and the President's Malaria Initiative (PMI) tend to focus on IRS, ITNs, and drug therapies. Large private donors prioritize new discoveries/technologies (e.g., vaccines). The recent call for malaria elimination is expected to result in changes in the selection of interventions, and additional research into uses of EM in varied settings is listed as necessary for improving vector control [54].

The survey did not assess EM impacts on other diseases. However, it is likely that the benefits would not be restricted to malaria. EM also has the potential to mitigate the incidence of diseases such as cholera and diarrhoea. In addition, EM can maximize the cost-effectiveness of other interventions (e.g., less amount of larvicide would be needed if mosquito breeding habitats located in drains were reduced). Donor support for initial massive drain cleaning and rehabilitation (especially in unplanned settlements), for preparation of community sensitization materials, and for increase in the number of garbage trucks is needed as part of a consorted effort toward the improvement of health and environmental conditions in the city. The Urban Sector Rehabilitation Project – USRP (1997–2004) and the Community Infrastructure Upgrading Programme – CIUP (2005–2008) are recent projects financed by the World Bank that included drainage construction [55,56]. However, coverage was restricted and health improvement was not an explicit goal.

## Conclusion

Results presented in this article provide initial evidence of the feasibility of EM for urban malaria control; they are based on a small-scale intervention that demands continued follow-up for a long-term evaluation of its sustainability, and that requires a detailed cost analysis. Still, after approximately 18 months of its implementation, the EM intervention revealed important lessons. The analyses suggested that four elements are needed for successful EM in democratic regimes: political will and commitment, community sensitization and participation, provision of financial resources for initial cleaning and structural repairs, and inter-sectoral collaboration. Failure to accomplish any of these four elements is likely to result in non-sustainable EM activities. The results here presented will, hopefully, foster discussions regarding community-based EM interventions, and catch the attention of donors on ways to promote activities that, at the same time, empower communities, improve health and environmental conditions, and ultimately contribute to poverty alleviation and sustainable development, as stated in the Agenda 21 [57].

## Competing interests

The authors declare that they have no competing interests.

## Authors' contributions

MCC planned Phases I and III of the EM activities, carried out all the data analysis, and wrote the manuscript. AT planned and implemented the sensitization programme (Phase II) and helped to draft the manuscript. SK and KK coordinated the implementation of EM activities and helped to draft the manuscript. SM helped to draft the manuscript. All authors have read and approved the final version of the manuscript.
